# Myocardial change with microvasculer obstruction after acute myocardial infarction

**DOI:** 10.1186/1532-429X-11-S1-P266

**Published:** 2009-01-28

**Authors:** Taniguchi Yasuyo, Takeshi Ishimoto, Shinitchiro Yamada, Sachiyo Iwata, Kazuo Mizutani, Akira Shimane, Takatoshi Hayashi, Teishi Kajiya

**Affiliations:** Himeji Cardiovasculer Center, Himeji, Japan

**Keywords:** Single Photon Emission Compute Tomography, Percutaneous Coronary Intervention, Acute Myocardial Infarction, Cardiovascular Magnetic Resonance, Acute Myocardial Infarction

## Introduction

Microvascular obstruction detected by Cardiovascular Magnetic Resonance (CMR) has been linked to ventricular remodeling and adverse cardiovascular events in acute myocardial infarction (AMI).

## Purpose

We analyzed how the changes of myocardium with MVO in human reperfused myocardium affect left ventricular function by using two imaging modalities, CMR and cardiac nuclear imaging.

## Methods

We investigated 28 patients with anterior AMI in whom primary percutaneous coronary intervention was performed successfully (mean age : 63.4 +- 8.4 year old, female 14.3%). From nuclear imaging, ejection fraction (LVEF), left ventricular volumes, and summed defect score (SDS) 2 weeks and 3 months post-MI were obtained by using rest gated technetium-99 m tetrofosmin electrocardiography-gated single photon emission computed tomography. Gdlinium contrast enhancement CMR imaging was performed within 2 weeks after AMI using a 1.5 T imaging unit. The patient's characteristics and cardiac parameters were compared between two groups: with MVO (group M, n = 18) and without MVO (group nM, n = 10).

## Results

Both groups had similar baseline characteristics about coronary risk factors. In group M, peak CK was higher (7208 IU/L and 3929 IU/L, P < 0.01) and reperfusion time was shorter (4.7 hr and 3.7 hr, P < 0.01) tnan group nM. Left ventricular end-diastolic volume (LVEDV) (142 ml and 134 ml, P < 0.01) and SDS (33 and 20, p < 0.05) showed significantly larger in early phase of AMI in group M. Although LVEDV at three months after AMI kept larger value (149 ml and 128 ml, p < 0.01) in group M, LVEF weren't different in each group. The improvement of LVEF was also remarkably larger in group M (21% and 9%). Any medication such as beta blocking agents and angiotensin converting enzyme inhibitors didn't affect these results. See Table 1.

**Table 1:** Cardiac performance

	group M		Group nM		p
		SD		SD	
EDV early (ml)	142	20	134	34	<0.01
ESV early (ml)	91	17	86	41	n.s.
EF(%)	37	5	40	13	n.s.
Follow up EDV (ml)	149	30	128	47	<0.01
Follow up ESV(ml)	86	24	70	42	<0.01
EF(%)	44	7	46	10	n.s.
SDS	33	10	20	14	<0.05

## Conclusion

Although MVO detected by CMR predict the ventricular remodeling even in successful coronary intervention, this adaptation is thought to increase cardiac output to some extent.

